# Amino acid biophysical properties in the statistical prediction of peptide-MHC class I binding

**DOI:** 10.1186/1745-7580-3-9

**Published:** 2007-10-29

**Authors:** Surajit Ray, Thomas B Kepler

**Affiliations:** 1 Department of Mathematics and Statistics, Boston University, Boston, MA, USA; 2 Center for Computational Immunology, Department of Biostatistics & Bioinformatics and Department of Immunology, Duke University Medical Center, Durham, NC, USA

## Abstract

**Background:**

A key step in the development of an adaptive immune response to pathogens or vaccines is the binding of short peptides to molecules of the Major Histocompatibility Complex (MHC) for presentation to T lymphocytes, which are thereby activated and differentiate into effector and memory cells. The rational design of vaccines consists in part in the identification of appropriate peptides to effect this process. There are several algorithms currently in use for making such predictions, but these are limited to a small number of MHC molecules and have good but imperfect prediction power.

**Results:**

We have undertaken an exploration of the power gained by taking advantage of a natural representation of the amino acids in terms of their biophysical properties. We used several well-known statistical classifiers using either a naive encoding of amino acids by name or an encoding by biophysical properties. In all cases, the encoding by biophysical properties leads to substantially lower misclassification error.

**Conclusion:**

Representation of amino acids using a few important bio-physio-chemical property provide a natural basis for representing peptides and greatly improves peptide-MHC class I binding prediction.

## Introduction

A key step in the development of an adaptive immune response to pathogens or vaccines is the binding of short peptides, to molecules of the Major Histocompatibility Complex (MHC) for presentation to T lymphocytes, which are thereby activated and differentiate into effector and memory cells. The rational design of vaccines consists in part in the identification of appropriate peptides to effect this process.

The task is complicated by the fact that genes of the MHC locus have some of the greatest allelic variability observed among functional loci [1]. Peptides that bind well to one allele may or may not bind well to another.

A variety of methods for predicting peptide-binding to specific MHC-alleles based on sequence information of the peptides have been developed. A comparative review of some of the most influential approaches, including Weight Matrix Models (WMM), Hidden Markov Models (HMM), and Artificial Neural Networks (ANN) can be found in [2].

Prediction algorithms can be categorized broadly into two main classes- those based on pattern recognition and those based on classification. Pattern recognition methods seek to discover similarities among the peptides that bind at high affinity to a given MHC allele (henceforth denoted as "binders"), without considering the properties of non-binders. On the other hand classification methods seek those characteristics that most effectively distinguish binders from non-binders. Pattern recognition-based methods include WMM and motif-based prediction and profile HMM [3,4],. Classification methods include Support vector machines [5] and Classification Trees [6-8]. These methods and the software implementing them are reviewed in [9] and [10].

Whether based on classification or pattern discovery, the peptides under investigation must have a representation in an appropriate space. The most commonly used prediction methods employ a simple categorical representation of the amino acids by chemical identity. In this representation, each amino acid is implicitly regarded as equidistant from every other amino acid.

Our aim in this paper is to determine the ability of more structured representations, based on the biophysical properties of the amino acids, with a goal toward improving the effectiveness of standard classification methods. Given that classification must always seek to balance parsimony, or simplicity in the model specification, against accuracy within the training set, a representation based on properties that may play a significant role in determining the binding characteristics of the peptide has a fair chance of supporting models that achieve accuracy with simple models.

For example, one property of amino acids that is clearly related to protein binding is hydrophobicity. The Kyte-Doolittle [11] hydrophobicity index induces an order on the amino acids. We may distinguish one set of amino acids, e.g., (R, K, D, E, N, Q, H, P, Y, W, S, T, G) from the remainder (A, M, C, F, I, L, V) by stating that the first set is to contain all amino acids with KD index less than that of A (Alanine). There are 21 ways to form such subsets, or log_2 _21 = 4.4 bits of information to specify the split based on the above ordered set. An arbitrary split into two groups, on the other hand, requires log_2 _2^20 ^= 20 bits for its specification. Note also that classification based on hydrophobicity when hydrophobicity is not strongly relevant can quickly become inefficient (such as "everything with KD index less than that of alanine, plus phenylalanine and valine; not including arginine, aspartic acid and tryptophan").

We do not intend to measure the information required for the specification of each classification model, but instead rely on the natural role of representational simplicity in the performance of classification methods. We may put it another way and ask whether a biophysical encoding makes it easier to find most of the binders by piling them up near each other in feature space, rather than having them scattered more diffusely at the level of individual residues.

To demonstrate the effectiveness of our feature space representation we compare the performance of several well-known classification methods under both a biophysical amino acid encoding and a simple categorical encoding. This paper does not focus on comparing the classification methods themselves. Instead, for each of the classification methods we compare the performance of the classifiers using the biophysical encoding against the usual categorical encoding.

In addition to effective classification, prediction methods based on amino acid biophysical properties may lend themselves naturally to the development of more comprehensive systems that combine purely empirical methods with *de novo *or first-principles prediction of peptide binding.

### Prior Art

In contrast to the substantial literature on sequence-based peptide binding prediction, there has been relatively little focus on the use of amino acid biophysical properties in binding prediction. Information about the amino acid properties can be used for prediction in several ways. One may, for example, use the real-valued properties themselves in a regression model, or more simply use the order induced by the properties. Alternatively, one can use these properties define new categorical variables and thus natural equivalence classes on the amino acids. [12] used statistical dissimilarity defined on "property models" which showed increased sensitivity over other existing methods. Using the public database on Amino Acid Properties [13], containing a total of 484 properties, [14] and [15] build prediction rules using SVM, decision trees and C4.5 and C5 [16,17]. [15] chose a list of 23 properties from different major and minor classes and measured the performance of classification algorithm based on specificity, sensitivity and accuracy. Additionally, the paper summarizes the three most important variables together with the most important positions for each of the MHC-I alleles they consider. On the other hand [14] started with all 484 properties (leaving out the 10 properties containing missing values) and used heuristic algorithm (based on the pairwise correlation coefficients among the properties) to remove redundancy. Finally, they report the misclassification error for C4.5, both with and without bagging, using all the variables that passed the redundancy test.

Using structural information, [18] describe a regression model to explain the binding affinity (*pIC*_50_) with the properties describing the 3-dimensional structure of the peptide. In particular [18]*pIC*_50 _regressed *pIC*_50 _values of peptides on two sets of position specific structural parameters, namely Isotropic Surface (ESI), area and Electronic Charge Index (ECI). Though none of regression coefficient themselves were statistically significant their approach provided a more desirable leave-one-out cross validation error. Another approach using amino acid biophysical properties has been proposed by [19]. They employ an encoding based on the biophysical properties to classify the 20 AA into four binary factors: Hydrophobic = {A, V, F, P, M, I, L}, Polar = {S, T, Y, H, C, N, Q, W}, Charged = {D, E, K, R} and Glycine = {G}. This coding assigns a corresponding *biochemical signature *to each peptide, where each position now belongs to a 4-letter alphabet rather than a 20-letter alphabet. Though this coding does not allow one to distinguish between amino acids with the same code, e.g., Leucine and Isoleucine, it gives a very important partition in reduced dimension, which is particularly relevant for peptide prediction. Using this dimension reduction [19] report better misclassification error compared to algorithms based on the full unstructured 20-symbol alphabet. Empirical evidence of superiority of property based methods has also been well documented in an array of recent literature including [20-26].

### Our approach

While many of the research work mentioned above examined the advantages of using bio-physio-chemical properties for MHC-peptide binding, often under particular classification frameworks, ours is the first article which provides the mathematical rigor of the generalized theory of representing the 9-mer amino acids into the space of amino acid properties. Our method was developed parallel to [14] and [15] and is closely related to their approaches. i.e. we use the full metric information of the amino acid properties, but we do not use any metric information of structural parameters. But one major difference to the approaches by [14] and [15] is that the properties we analyze are first screened on the basis of their importance based on X-ray crystallography study of peptide binding phenomenon reported in the literature. This screening is based on the crystallographic study, rather than being completely determined by data from AAindex. This step is extremely important, for several reasons. First, the values of AA properties listed in the AAindex are based on experimental data, which are not standardized and often results in discrepant measurement of the same property. Moreover, there exist a lot of redundancy e.g. the database contains three indices, one each for negative, positive and net charge. There are also instances of one index being a more precise version of another index e.g. Electron-ion interaction potential by [27] and [28]. Finally the properties chosen by exhaustively searching the AAindex is time consuming and often may not be easily interpretable. Our screening of AA properties based on their relevance in binding avoids these difficulties.

The main goal of this paper is to show that by starting with a small set of properties known a priori to be of importance in protein-protein binding, and then by using statistical techniques for variable selection to further refine this set of properties, leads to a significant decrease in the misclassification error compared to simple sequence-based classification. Moreover, as our starting set is known a priori to be relevant in MHC binding, the final subset of properties can be directly interpreted and later used to formulate *de novo *or first-principles prediction of peptide binding. Finally, to our knowledge this is the first research comparing sequence-based and property-based classification of MHC-binding peptides using a number of competitive classification algorithms.

The layout of this article is as follows: the Methods section describes the steps used in choosing the biophysical properties. The Results section presents a direct application of the proposed algorithm on a training peptide binding dataset for MHC allele A*0201, which has been previously used by [2] to compare several sequence-based classification algorithms. The last section provides a detailed discussion and comparison with competing methods.

## Methods

This section describes the steps used in simultaneously choosing the important biophysical protein properties that govern the peptide-MHC binding and providing a classification scheme based on these properties.

### Amino acid properties

The first step toward implementing our proposed method is to collect data on those properties that are hypothesized in the literature to play an important role in the MHC-peptide binding. Using the extensive literature in X-Ray crystallography of MHC molecules e.g. in [29-35] available on several MHC-alleles, together with a substantial literature on structural correlates of MHC-binding e.g. in [36-39], there is opportunity to assemble a set of properties to serve as our starting set. Given a particular MHC-allele, the MHC-peptide binding is mainly determined by the peptide's back-bone conformation and the interaction of the side-chains with the MHC-binding grooves [36]. It is well known that structural properties for different MHC alleles differ considerably, and thus the set of screened properties may be different for different alleles. On the other hand, though the structural details may differ, e.g. the specific anchor positions may differ, the properties governing binding may remain the same. So in this paper we propose to use a common set of properties determined from [29-32] as the starting set. It should be noted that as our approach includes a model selection step, the final result is independent of the initial set of properties chosen, as long as the set contains all the important properties. So while choosing the starting set we should make a liberal choice and allow the model selection step to choose the final set.

### Classification Algorithms

Our methodology is not restricted to any particular classification tool, rather it is a general model-selection technique applicable to any classification tool. Note that most of the biophysical properties that are important either lie on the real line ℝ or are indicator variables. So unlike the original amino acid representation, we are not restricted to using special classification tools designed or adapted for categorical data, rather we can use a larger class of classification tools available for continuous data

### Variable selection

For any specific classification algorithm one crucial step is to select the set of variables which gives us the best parsimonious classification rule. This selection can be performed using two different models- one in which we select the same properties for each positions in the peptide, and the other in which we allow different properties for different positions. Both have their merits and demerits. In this paper we will mostly deal with the first model, in which all the positions are restricted to select the same properties, but it can be easily generalized to the unrestricted form of position specific variable selection.

The purpose of the variable selection step is twofold. On one hand the selected properties should help us design the most parsimonious classification rule based on the training data. On the other hand this step can serve as an exploratory tool, which would provide a quantitative basis for narrowing down on previously unknown peptides, on which laboratory experiments can be performed. (Note: At present, motifs are the basis of narrowing down on possible binders). To illustrate this, let us look at the binding motifs of the set of available binders and non-binders for allele HLA*0201.

**Figure 1:**
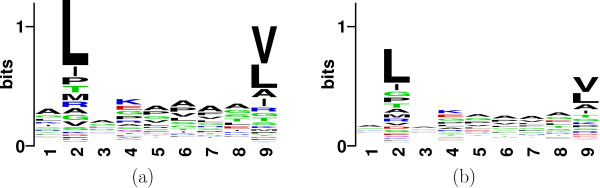
Sequence Logo plot of position specific conservation of (a) binders and (b) non-binders to HLA-A0201.

Figures 1(a) and 1(b) describes the sequence logo plots of available binders and non-binders to allele A*0201 (database details in section 4). It can be hypothesized that the non-binders included in this dataset were initially investigated because they were considered as be potential binders based on their motif resemblance with existing binders. Thus, classification on this dataset provides a particularly stringent test.

For any particular classifier we start with the initial set of properties and employ the forward selection method using misclassification error as the criterion to choose a subset. Starting with each single property we calculate the average of misclassification errors of 10 runs of a 10-fold classification and choose the property which gives the lowest misclassification error. In the second step, we keep the property selected in the first step and append one property at a time from the previously unselected properties and choose the second property as the pair (with the first property chosen) with lowest misclassification error. This process is continued until their is no gain in misclassification error by adding a new variable. Note that we choose the forward selection algorithm for computing speed but one can also use backward selection or step-wise selection algorithm.

## Results and Implementation

The algorithm is demonstrated with HLA-A*0201 binding peptides generously provided by V. Brusic. His group used this data in a comparative analysis of prediction methods [2]. This dataset consists of 1146 peptides, of which 359 have been experimentally verified to bind to HLA-A*0201. The remainder have been shown experimentally to fail to bind to HLA-A*0201. The original source of much of this data is the MHCPEP [40], which further subclassifies the binders as High, Medium, and Low binders. But as laboratory methods and conditions are not yet standardized, binding affinities are not always reliable. So in this paper we choose to use the binary classification of these 1146 peptides.

### Amino Acid Properties Chosen

Based on the literature survey we start with the following properties Molecular Weight, Volume, Area, Hydrophobicity, Isoelectric point and the indicator variables aliphatic, aromatic, branch and sulphur.

### Classification Software

In this paper, we will demonstrate our method using three classification algorithm, namely Support Vector Machines [5], Random forest [8] and Bagging [7]. We choose this three algorithms for two specific reasons. First, these three algorithms are quite different and thus have different optimality properties. Second, it serves the purpose of comparing with the sequence-based classification results reported in [2]. Note that the above classifiers are not especially designed in the context of MHC-peptide binding prediction. We expect to see the same relative improvement for classifiers, which are designed especially for predicting binders to MHC molecules.

All analyses are done using freely distributed R package [41] and contributed packages in the R development website. Variable selection and comparison codes are available from the authors upon request.

• **Support Vector Machine (SVM)**: Using the R package e1071. SVM was performed with the default "Gaussian" kernel. To be more specific the kernel was given by

*k*(*u, v*) = exp(-γ*|*u*-*v*|^2^) where *γ *= 1/(data dimension)

• **Random Forest (RF)**:Using the R package randomForest and using the default values.

• **Bagging (BAG)**:Using the R package ipred and again using the default values.

### Variable Selection results

Starting with the set of properties listed in Section, we now employ the forward selection algorithm described in Section Table 1 gives the steps in variable selection and the corresponding misclassification error.

**Table 1:** Selected Variables for each of the three classifiers using available binding data for MHC Class I allele A*0201

Classifier	Step	Variable Selected	Misclassification error	Gain Achieved
SVM	1	hydrophobicity	0.171839	
	2	Volume	0.142022	0.0298169
	3	isoelec	0.125781	0.0162415
	4	branch	0.118570	0.0142109
	5	aromatic	0.118395	0.0001743
Random Forest	1	isoelec	0.136791	
	2	Volume	0.130078	0.0067131
	3	hydrophobicity	0.129642	0.0004359
Bagging	1	hydrophobicity	0.146033	
	2	Area	0.140279	0.0057541
	3	isoelec	0.137227	0.0030514
	4	aromatic	0.134786	0.0024411

SVM selects Hydrophobicity as the most important property with a corresponding 10-fold CVM of 0.172. Keeping the variable Hydrophobicity, we next explore the remaining variables to find out the next important variable which gives the best CVM, subject to the condition that corresponding CVM is less than 0.172. Using this guideline we next choose Volume and using Volume and Hydrophobicity the corresponding CVM decreases to 0.142. Following the same criterion we then include Isoelectric point, branch and aromatic in the given order as the 5 most important variable for the SVM classifier with corresponding CVMs of 0.126, 0.1185 and 0.1183. The next best variable chosen actually increased the CVM, so we stop the process after choosing the 5 variables. Following the same steps, random forest chooses Isoelectric point, Volume and Hydrophobicity in the given order as the 3 most important properties. Including a fourth variable from any of the remaining variables actually increased the CVM. Again following the same selection steps and stopping rule, we choose Hydrophobicity, Area, Isoelectric point and Aromatic as the four most important variables for Bagging.

Variable selection results indicate that different properties are important for different methods, but there is an overall consensus about the importance of the three properties Hydrophobicity, Volume (though bagging does not select Volume, Area is highly correlated with Volume) and Isoelectric point. The discrepancy in the order and final set of selected variables can be well explained by the difference in steps, optimizer and the way variables enter the classifiers. For example while SVM uses the full metric information of the properties, random forest and bagging, which are essentially tree based methods, use the metric values for partitioning the space, thereby using only the ordering induced by the property.

Other interesting observations from this analysis are

• The gain from adding new properties is more significant in SVM than in bagging and Random Forest. Again the reason might be that as RF and bagging does not use the metric values of the properties, the extra information on partitions provided by including any new property may not be significant, where as for SVM the increment is more significant.

• Within the given set of properties SVM achieves the lowest misclassification error with 5 properties (Hydrophobicity, Volume, Isoelec, Branch, Aromatic, where the last two variables are indicator of whether ....), as well as among all 3 property based classifiers SVM achieves the lowest misclassification error of 0.125781, using Hydrophobicity, Volume and Isoelectric point.

In summary, we can conclude that the properties selected as the most important in predicting the MHC-peptide binders are considerably robust with regards to our choice of classifiers and moreover these properties are well understood. For HLA A*0201 we have chosen Hydrophobicity, Volume and Isoelectric point and Table 2 lists the values of these properties for the 20 amino acids. For predicting a new peptide, we first represent the sequence in the space of the three chosen property, e.g. a 9-mer peptide sequence will be represented as a 27 dimensional numerical vector and then we will use the classifier trained on available training data to classify the new peptide. Generalization to testing the peptide binding affinity against more than one allele is a trivial, keeping in mind that the final set of properties which gives the best CVM might well be different in size and constitution, i.e. for a different allele we may choose a different set of properties.

**Table 2:** Values of three most important indexes (properties) of amino acids determining the peptide-MHC binding Reproduced from [43]^1^, [11]^2 ^and [44]^3^

1L	Name	Volume^1^	Hydrophobicity^2^	Isoelectric^3^
A	alanine	88.6	1.8	6.00
C	cysteine	108.5	2.5	5.05
D	aspartate	111.1	-3.5	2.77
E	glutamate	138.4	-3.5	3.22
F	phenylalanine	189.9	2.8	5.48
G	glycine	60.1	-0.4	5.97
H	histidine	153.2	-3.2	7.47
I	isoleucine	166.7	3.8	5.94
K	lysine	168.6	-3.9	9.59
L	leucine	166.7	3.8	5.98
M	methionine	162.9	1.9	5.74
N	asparagine	114.1	-3.5	5.41
P	proline	112.7	-1.6	6.30
Q	glutamine	143.8	-3.5	5.65
R	arginine	173.4	-4.5	11.15
S	serine	89.0	-0.8	5.68
T	threonine	116.1	-0.7	5.64
V	valine	140.0	4.2	5.96
W	tryptophan	227.8	-0.9	5.89
Y	tyrosine	193.6	-1.3	5.66

### Comparison of Property-based and Sequence-based classifiers

In this section we provide a comparison of our newly designed property-based classifiers with the sequence-based classifiers. As noted earlier we will perform the comparison by evaluating the misclassification error under different representation (property based or sequence-based) for the different classification schemes.

To illustrate the performance of using property based classification, we will focus on the three properties Hydrophobicity, Volume and Isoelectric point and their composite effect. Note that there may be some properties that are highly correlated to one of the three selected properties (e.g. Molecular Weight and Area are very highly correlated with Volume), and may perform equally well. But, the purpose of this section is to show that the appropriately chosen properties, we can build efficient prediction algorithm which are substantially better than the sequence-based classifiers.

**Figure 2:**
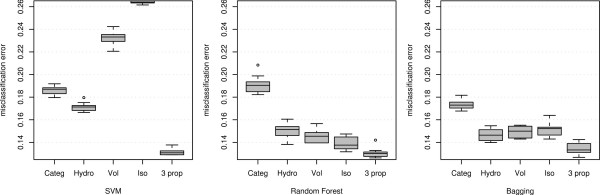
Misclassification error using different variables and classification methods applied to the MHC binding data for Class I allele A*0201.

Figure 2 illustrates the misclassification errors for 100 runs of 10 fold cross-validation under the three classifiers SVM, Random Forest and Bagging. The five boxes under each method represent the misclassification error under five representations namely

(i) Original Amino Acid Coding (Categ)

(ii) Hydrophobicity values (Hydro)

(iii) Volume (Vol)

(iv) Isoelectric Charge (Iso)

(v) All three properties above (3 prop)

Again we specify that all these classifiers use the default tuning parameters available in the respective R-packages.

It can be observed that for all three classifiers the three-property-based classification outperforms the performance of the sequence-based classification. It is also interesting to note that for each of the classifiers, the single most important variable chosen through minimization of CVM alone performs better than the sequence-based classification. Moreover, for RF and BAG any single property achieves lower misclassification than the sequence-based methods. The last observation can again be supported by the fact that RF and BAG uses the metric values to order the amino acids and we can infer that any single property alone provides a better ordering for predicting the binding affinity than the full information provided by the actual amino acid sequence of the peptide.

### Comparisons using AROC

One common criticism of using misclassification rate as a measure of comparison is its dependence on tuning parameters of the specific classifiers. So, for comparing the performance of the property based and sequence-based classifiers we use the criterion of Area under the Receiver Operating Characteristic curve (AROC). The ROC plots the sensitivity vs specificity for a range of tuning parameters chosen for each of the classifiers. We calculate the AROC as a numerical approximation for the area under the ROC curve. This AROC value is a global measure and it ranges between 1 and .5, where the value of 1 corresponds to perfect classification and the value of .5 corresponds to random classification of an observation into one of the two classes. The AROC will, on one hand, enable us to compare the performance of different representations (sequence-based and property based) for the same classifiers and, on the other hand, as it is a global measure, we can compare our classifiers with other competitive methods.

**Figure 3:**
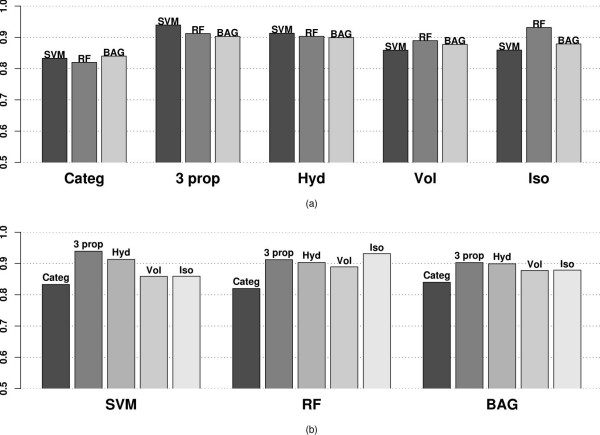
AROC values using different variables and classification methods applied to the MHC binding data for Class I allele A*0201 categorized by (a) variables used (b) classifier.

The Area under the Receiver Operating Characteristic (AROC) [42] for the three classifiers using 5 different amino acid representations as in the previous subsection are displayed in Figure 3. Panel (a) represents the AROC values grouped by representations whereas panel(b) represents the same AROC values grouped according to the classifiers

The AROC values demonstrate the same trend as the misclassification error box plots in Figure 2. SVM performs best using three properties (0.939), followed closely by RF using only Isoelectric point (0.932) and SVM using Hydrophobicity also (0.9132) alone. The AROC values represent the same trend as the misclassification error, which indicates that the improvements gained using our method are not sensitively dependent on the choice of tuning parameters.

In general, these findings demonstrate how the property-based methods perform considerably (misclassification error .90 to .94) better than the sequence-based method under these three classifiers (misclassification error .81 to .84). Though these classifiers (SVM, RF and Bagging) are off-the-shelf methods (not especially designed for biological sequence analysis), we observe that the misclassification errors are in the same range as the ones analyzed by [2]. For the six classifiers (Bimas, Syfpeithi, Artificial Neural network, HMM, and two developed by the authors namely YK0201, YKW0201) considered by [2] the misclassification error ranged from (.81 to .87), the upper range slightly higher than the misclassification error of our sequence-based misclassification errors. But our off-the-shelf property-based classifiers perform even better than these specially-designed classifiers which are based on the sequence. Though we do not make a direct comparison with other classifiers, this study provides enough evidence that any classifier may perform better with carefully chosen properties.

## Discussion

In spite of great successes in the amelioration of infectious disease, new threats, such as SARS and avian influenza continue to arise, and old foes, such as malaria and polio, resurge. In the future, we have to learn to make vaccines that induce better protective immunity than natural infection is capable of doing. One possible direction is to move toward epitope-based vaccines.

In spite of large quantities of new data, there is still a major role to be played by prediction, since there are 20^9 ^peptides and more than 1000 MHC alleles. The vast majority of the literature in this field have focused on developing empirically derived techniques, but our approach in this paper provides a single technique to a general methodology and paves the way for further development based on the statistical infrastructure. The property space we propose is designed to effectively capture higher level motif interaction of the binding phenomenon and thus provide the framework for testing scientific hypothesis of peptide-binding phenomenon. The field of peptide binding prediction has matured and now we are faced with huge amounts of binding data on previously unexamined peptides and we believe that the techniques described in this paper will provide the rich mathematical and statistical basis for in-silico binding predictions.
